# An Exploratory, Retrospective Study of the CYPRI Score for Pharmacogenetic Testing Among Mental Health Patients

**DOI:** 10.3390/genes16121479

**Published:** 2025-12-09

**Authors:** Samira Marie Comtesse, Ivana Tašková, Nicole Safářová, Martina Hahn

**Affiliations:** 1 Department of Psychiatry, Psychosomatics and Psychotherapy, University Hospital, Goethe University Frankfurt, 60528 Frankfurt, Germany; 2 Department of Clinical Pharmacy, Psychiatric Hospital Bohnice, 18201 Prague, Czech Republic; 3 Department of Social and Clinical Pharmacy, Faculty of Pharmacy in Hradec Králové, Charles University, 50003 Hradec Králové, Czech Republic; 4 National Institute of Mental Health, 25067 Klecany, Czech Republic; 5 Third Faculty of Medicine, Charles University, 10000 Prague, Czech Republic; 6 Department of Pharmacology and Clinical Pharmacy, Philipps-University, 35037 Marburg, Germany; 7 Department of Mental Health, Varisano Hospital Frankfurt Hoechst, 65929 Frankfurt, Germany

**Keywords:** PGx, CYPRI Score, impact, pharmacogenetics, pharmacotherapy, personalized medicine

## Abstract

**Background/Objectives**: Pharmacogenetic (PGx) testing is gaining importance in optimizing psychiatric pharmacotherapy, yet routine use remains limited due to cost and unclear patient selection criteria. The CYP Pharmacogenetic Risk Score (CYPRI) is a clinical tool designed to identify psychiatric patients most likely to benefit from PGx testing, based on medication profile, adverse drug reactions (ADRs), and therapeutic drug monitoring (TDM) results. This study aimed to evaluate the clinical relevance of the CYPRI by identifying its weaknesses and gaps in a clinical setting, propose targeted modifications to address those limitations, and assess the applicability of the improved version in a routine clinical setting. **Methods**: In a retrospective analysis, data from 92 patients with depression at Frankfurt University Hospital were evaluated using the CYPRI score. Its association with the clinical impact of PGx testing, measured by the IMPACT score, was analyzed using ordinal regression and Receiver Operating Characteristic (ROC) analysis. Based on the findings, a revised version of CYPRI was developed and applied to the retrospective cohorts of Frankfurt and Prague. **Results**: The original CYPRI score was significantly associated with increased IMPACT score, suggesting its clinical value in detecting non-normal CYP2D6 and/or CYP2C19 metabolizers. However, the corrected version (hereafter referred to as CYPRI_cor), which emphasized clinically relevant pharmacokinetic factors, showed improved clinical specificity while maintaining similar discriminative performance. In the Frankfurt cohort, the area under the curve (AUC) for CYPRI_cor was 0.68 (95% CI 0.56–0.79), and in the Prague cohort, the AUC for CYPRI_cor was 0.71 (95% CI 0.60–0.81). While the overall discriminative ability in Frankfurt was slightly lower, CYPRI_cor achieved a specificity of 0.69, enabling more precise identification of patients most likely to benefit from PGx testing. A CYPRI Cut-off of ≥4 was determined to indicate clinical impact. **Conclusions**: The CYPRI_cor score was designed to optimize and to rule out potential limitations of the original score, particularly regarding the attribution of ADRs and the weighting of TDM results. Although the modifications did not improve discriminative performance in the Frankfurt dataset, the proposed changes remain meaningful. Prospective clinical studies need to verify the clinical utility of the CYPRI_cor.

## 1. Introduction

Pharmacogenetic (PGx) testing is becoming increasingly relevant in the clinical-pharmacological treatment of patients as it emerges as an effective method of personalized medicine, offering clinicians the ability to optimize drug therapy based on individual genetic profiles. In psychiatric pharmacotherapy, where treatment response and tolerability vary widely among patients, PGx testing holds particular promise to personalize treatment strategies and guide medication decisions [1,2,3].

Pre-emptive genotyping enables the identification of genetic variants that influence individual drug response. Such testing can reduce the risk of adverse drug reactions (ADRs) linked to genetic predisposition, promote faster remission through shorter hospital stays, and improve overall treatment efficacy by increasing the likelihood of a positive therapeutic response [4,5,6].

Despite these benefits, routine implementation of PGx profiling has yet to be established in German clinical practices [7]. The costs associated with genotyping remain a barrier to the routine implementation of PGx testing in everyday clinical practice, as well as the limited awareness among physicians and insufficient expertise in interpreting PGx results [8]. As a result, there is a growing need for tools that can help identify those patients who are most likely to benefit from such testing, thereby ensuring the efficient and targeted use of available resources.

PGx testing is also gaining importance in the field of psychiatry. Several clinical studies have demonstrated associations between specific genetic polymorphisms and the therapeutic response to antidepressants [1,9]. Major depressive disorder (MDD) affects more than 260 million people worldwide and remains one of the most common and burdensome mental health conditions globally. However, pharmacological treatment of depression remains challenging [1]. Current treatment strategies predominantly follow a trial-and-error approach, in which the selection of effective medications is based primarily on empirical judgment and gradual adjustments [10]. This results in remission rates of only about 30% during first-line therapy [11]. Investigating gene–drug interactions, especially regarding antidepressant metabolism, has the potential to improve treatment outcomes [12].

Notably, an estimated 87% of patients with depression carry at least one genetic variant that could influence their response to commonly prescribed psychotropic medications [13]. Many antidepressants and antipsychotics are metabolized by the polymorphic cytochrome P450 (CYP) enzymes CYP2C19 and CYP2D6 [1]. CYP2D6 and CYP2C19 are hepatic enzymes of the cytochrome P450 family that play key roles in drug metabolism. CYP2D6 metabolizes around 20% to 25% of clinically used drugs, while CYP2C19 contributes to the metabolism of drugs such as clopidogrel, voriconazole, proton pump inhibitors, antidepressants, carisoprodol, and diazepam, as well as endogenous compounds like melatonin and progesterone. Genetic variations in CYP genes that encode drug-metabolizing enzymes can cause significant differences in drug response and adverse effects [14,15]. Accordingly, international guidelines provided by the Clinical Pharmacogenetics Implementation Consortium (CPIC) and others provide genotype-based dosing recommendations for several psychiatric medications [16]. The Dutch pharmacogenetic working group (DPWG) even recommends preemptive testing before starting certain antidepressants [17].

However, the question remains in times of declining financial resources in the health care system: which patient should be tested? Considering financial and logistical constraints, a systematic and clinically grounded method for identifying high-risk patients is needed to ensure that PGx testing is applied where it offers the greatest benefit.

The CYP Pharmacogenetic Risk Score (CYPRI) is a novel clinical decision-support tool developed at the Psychiatric Hospital Bohnice in Prague (Czech Republic). It aims to address this need by providing a structured framework to assess the likelihood that a given mental health patient would benefit from PGx testing. The score integrates key clinical risk factors, including medication profiles, ADR, therapeutic drug monitoring (TDM) results, and treatment resistance, to estimate a patient’s risk of being an altered metabolizer phenotype of CYP2D6 or CYP2C19 substrates.

In the context of precision psychiatry, CYPRI is meant to close an implementation gap created by largely unselected testing. This approach is intended to support, not replace, clinical judgment by providing a structured way to prioritize patients for PGx testing in psychiatric care. Grounded in routine clinical practice, CYPRI has shown consistent associations between higher scores and greater medication impact from PGx results, underscoring its potential to enable more targeted and resource-conscious implementation [18].

The aim of this study is to retrospectively apply the CYPRI Score to clinical data from 92 psychiatric patients treated at the University Hospital Frankfurt, testing its clinical relevance and providing the first reproduction and external application of the score in a healthcare setting outside the original development center in Prague. By analyzing discharge summaries and pharmacotherapeutic documentation, the study evaluates the practical utility of the CYPRI Score in real-world clinical settings. Specifically, the goal is to assess its effectiveness in identifying patients with clinically relevant *CYP2D6* and/or *CYP2C19* polymorphisms and to correlate CYPRI scores with subsequent medication-related interventions. Building on the initial version (CYPRI_orig), we introduced a corrected version (CYPRI_cor) that specifies the ADRs to include only pharmacokinetically plausible adverse reactions and refines TDM scoring with a more individualized scoring system.

In doing so, this study increases the utility of the CYPRI Score as a potential decision-support tool for PGx testing in psychiatry. Furthermore, the results allow for a critical appraisal of the score’s applicability, including its limitations and possible avenues for refinement to enhance its clinical value and usability.

## 2. Materials and Methods

### 2.1. Study Design and Patient Population

This retrospective, observational study is based on metabolizer phenotype information of patient data collected during a previous research project, FACT-PGx (ethics approval 2021/138), conducted at Goethe University Frankfurt. Between July 2021 and January 2022, all patients over the age of 18 who were electively admitted for treatment of a depressive disorder (ICD-10: F32.x and F33.x) to one of the two open depression wards of the Department of Psychiatry, Psychosomatics and Psychotherapy at University Hospital Frankfurt were offered genotyping for *CYP2D6* and *CYP2C19*. All additional clinical variables required for CYPRI scoring were extracted from clinical records of the patients.

Following written informed consent, 104 patients underwent genotyping. For the present analysis, 92 patients with complete genotyping results and accessible clinical documentation were included. Eleven patients had to be excluded due to a restriction on accessing their medical records, which had been sealed at the patient’s request.

In addition to the Frankfurt dataset with n = 92, the CYPRI_cor was also applied again to the cohort (n = 39) from the psychiatric hospital Bohnice in Prague (ethics approval PNBek7/2024), where the original CYPRI score was tested upon. The original CYPRI publication cohort was recruited between 31 January 2024 and 15 May 2025 and included patients who fulfilled at least one CYPRI criterion. PGx testing was typically recommended for treatment-resistant conditions, unexpected therapeutic drug monitoring findings, or clinically relevant adverse drug reactions at standard or low doses.

To ensure consistency and comparability, CYPRI scoring in both cohorts was performed by the same raters.

### 2.2. Genotyping Procedure

Biological samples of the Frankfurt and Prague cohorts were collected during hospitalization and sent to a certified external laboratory for analysis. Polymorphisms of the genetic disposition for the *CYP2D6* (*1, *2, *3, *4, *6, *9, *10, *14, *17, *34, * 35, *39, *41, *46, *58, *64, *69, *71, *82, *88, and *114) and *CYP2C19* (*1, *2, *3, *4, and *17) were analyzed. Genotyping of relevant gene variations in *CYP2D6* single-nucleotide polymorphisms (SNPs) and star allele coverage was performed on a MassArray Analyzer 4 system (Agena Bioscience GmbH, Hamburg, Germany) based on a self-designed panel using SpectroCHIP^®^-96 Arrays and the iPLEX^®^ Pro chemistry following the instructions supplied by the manufacturer. Due to specificity issues, a restriction fragment length polymorphism method was performed to determine rs3892097, rs16947, and rs1080995. Primer sequences are available on request. Moreover, copy number variations (CNV) were determined using the *CYP2D6* RealFast™ CNV Assay, provided by ViennaLab Diagnostics GmbH, Vienna, Austria (ViennaLab Diagnostics GmbH. CYP2D6 RealFast CNV Assay 2021). The laboratory was certified by a quality control program (INSTAND Gesellschaft zur Förderung der Qualitätssicherung in medizinischen Laboratorien e. V. 2020. Accessed on 10 February 2020). Haplotypes were defined for all analyzed SNPs according to gene-specific haplotype tables from the PharmVar homepage [19]. Phenotypes were assigned according to CPIC: for CYP2D6, poor (PM), intermediate (IM), normal (NM), or ultrarapid (UM) metabolizer (no RM category); for CYP2C19, poor (PM), intermediate (IM), normal (NM), rapid (RM), or ultrarapid (UM) (accessed 8 March 2022) [20,21].

### 2.3. CYPRI Score Assessment

To identify patients most likely to benefit from PGx testing, the CYPRI_orig was retrospectively applied. This structured scoring system was developed at Psychiatric Hospital Bohnice in Prague in 2024 and designed to detect clinical patterns suggestive of altered drug metabolism via CYP2D6 or CYP2C19.

The scoring system integrates five clinical domains known to be associated with altered CYP metabolism:

1. The use of CYP2C19/CYP2D6-relevant psychiatric medication.
2. The use of CYP2C19/CYP2D6-relevant somatic medication.
3. Documented treatment resistance despite confirmed adherence and adequate dosing.
4. Striking therapeutic drug monitoring (TDM) results.
5. Clinically significant adverse drug reactions (ADRs).

The presence of each criterion contributes either 1 or 3 points to the total score, depending on its estimated predictive value for detecting non-normal metabolic phenotypes. The total CYPRI Score ranges from 0 to 9, with higher scores indicating an increased likelihood of clinically relevant pharmacogenetic findings.

ADRs were scored only when a clinically plausible pharmacokinetic link was evident from the documentation, and clinically relevant drug–drug interactions were excluded using the Flockhart interaction tables [22]. Ambiguous or missing ADR information was assigned 0 points, and missing TDM data also received 0 points.

An overview of all CYPRI criteria is presented in Table 1.

#### 2.3.1. CYPRI Score Adjustment

Secondly, a corrected version of CYPRI_orig (CYPRI_cor) and IMPACT_orig was applied and compared in both cohorts.

1. ADR scoring adjustment

ADRs that could not be clearly linked to pharmacokinetic variability, such as weight gain or non-specific dermatological reactions (e.g., rash), were excluded from the 3-point ADR category. This decision was supported by clinical relevance considerations and guided by established adverse effect rating tools [25,26].

2. TDM scoring adjustment

The original 3-point scoring for abnormal TDM results was subdivided into more nuanced subcategories:

- 3 points if the patient is out of the therapeutic range.
- 1 point if the metabolic ratio MPR is out of range, but plasma level is within range.
- 1 point if the dose-corrected ratio C/D is out of range.

Table 2 illustrates the changes. After implementing these modifications, the CYPRI_cor score was also applied to the original dataset of 39 psychiatric patients from Psychiatric Hospital Bohnice in Prague, who were included in the initial development of the CYPRI scoring system. This secondary application aimed to assess whether the proposed refinements improved clinical discriminatory power across different patient populations and methods of information collection, namely closed retrospective documentation review in the Frankfurt cohort versus active scoring during hospitalization in the Prague cohort, where information could still be retrieved from ongoing records or supplemented by interviews.

#### 2.3.2. IMPACT Score Assessment

Table 3, describing the IMPACT_orig score, provides a standardized framework for assessing the clinical impact of pharmacogenetic findings and which was used as the primary outcome to benchmark CYPRI performance.

### 2.4. Statistical Analysis

To evaluate the predictive performance of the CYPRI and IMPACT scoring systems, a multistep statistical analysis was conducted. Given the ordinal nature of the outcome variable (IMPACT score), ordinal logistic regression was applied to assess the association between total CYPRI scores and the likelihood of pharmacogenetically driven clinical interventions. The model’s assumptions, including the proportional odds assumption, were verified prior to interpretation.

To examine the discriminative capacity of the CYPRI score, a Receiver Operating Characteristic (ROC) analysis was performed. The AUC was calculated to quantify the score’s ability to distinguish between patients with major PGx-related medication impact (IMPACT_orig ≥ 2) and those with minimal or no pharmacogenetic implications (IMPACT_orig = 0–1.5). The optimal threshold for clinical application was determined using Youden’s Index, which maximizes the sum of sensitivity and specificity. Internal validation of the main model (Frankfurt cohort, model CYPRI_cor IMPACT_orig) was performed using non-parametric bootstrapping with 1000 resamples. In addition to the ROC analysis, ordinal logistic regression models were applied to assess the relationship between CYPRI scores (both original and corrected versions) and the degree of pharmacogenetic impact (IMPACT score). The proportional odds model with a logit link function was used, implemented in the MASS package in R. Results are reported as odds ratios (ORs) with corresponding 95% confidence intervals (CIs) and *p*-values.

All statistical analyses were performed using R software version 4.2.2 (R Foundation for Statistical Computing) within RStudio version 2025.05.0+496 (Posit Software, PBC), and *p*-values below 0.05 were interpreted as statistically significant. No adjustments for multiple testing were made due to the exploratory nature of the study.

### 2.5. Use of Generative AI

No generative artificial intelligence (GenAI) was used in the generation, analysis, or interpretation of data. Textual formulation was manually conducted by the authors, with minor support in language refinement when necessary. Language refinment was supported using ChatGPT (OpenAI), version GPT-4.1.

## 3. Results

### 3.1. Study Population Characteristics

#### 3.1.1. Frankfurt Cohort

The study sample initially consisted of 104 patients who had consented to pharmacogenetic testing. The average age was 43 years (SD ± 15.1), with a median of 43 years. The majority of patients (51%) were between 18 and 39 years old. The gender distribution was balanced, with 52 female (50%) and 52 male (50%) patients.

For the present analysis, clinical and genotyping data from 92 patients were available and included. Eleven patients had to be excluded due to a restriction on accessing their medical records, which had been sealed at the patient’s request.

#### 3.1.2. Prague Cohort

In addition to the Frankfurt cohort, the CYPRI_cor score was also applied to a sample of 39 patients treated at Psychiatric Hospital Bohnice in Prague, Czech Republic. This cohort consisted of 29 inpatients and 10 outpatients with a range of psychiatric disorders. The mean age was 37 years (range: 17–70), and the majority of patients were male (26 men, 67%; 13 women, 33%).

### 3.2. Results Using the CYPRI_orig and IMPACT_orig Score in the Frankfurt Cohort

**Figure 1:**
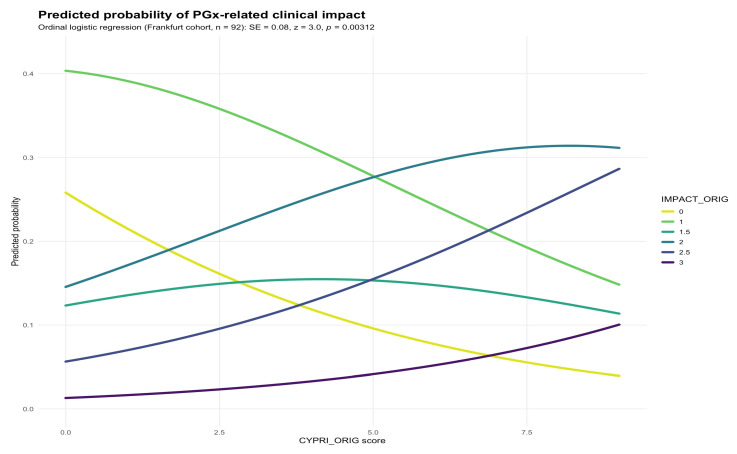
Predicted probability of IMPACT_orig by CYPRI_orig in the Frankfurt cohort (n = 92).

To evaluate the performance of the original CYPRI scoring system, we examined the association between the total CYPRI score and the clinical outcome (IMPACT) in the Frankfurt sample (n = 92). Ordinal logistic regression revealed a statistically significant association between higher CYPRI scores and greater PGx-related clinical impact (SE = 0.08, *p* = 0.00312; Figure 1). This suggests that an increasing CYPRI score is associated with a higher likelihood of a clinically meaningful impact on medication (e.g., medication adjustment or increased monitoring).

**Figure 2:**
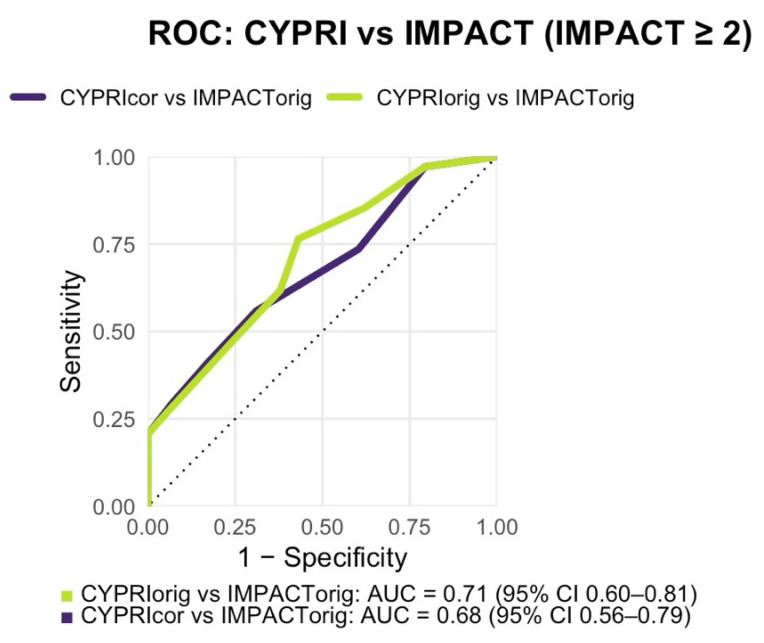
Receiver Operating Characteristic (ROC) analysis comparing CYPRI_orig vs. IMPACT_orig (AUC = 0.71, 95% CI 0.60–0.81) and CYPRI_cor vs. IMPACT_orig (AUC = 0.68, 95% CI 0.56–0.79) in the Frankfurt cohort (n = 92).

In addition, a Receiver Operating Characteristic (ROC) analysis (Figure 2) was conducted to assess the discriminative performance of the CYPRI score in identifying patients who experienced major medication impact (defined as IMPACT score ≥ 2). The resulting area under the curve (AUC) for CYPRI_orig was 0.71 (95% CI 0.60–0.81; Figure 2), indicating acceptable discriminatory power of the score in this clinical setting. Internal validation by non-parametric bootstrapping with 1000 resamples yielded a mean AUC of 0.60 (95% CI 0.48–0.70), consistent with the DeLong estimate and indicating moderate internal stability of the model performance.

Using Youden’s Index, the optimal CYPRI cut-off for identifying patients with significant PGx impact was determined to be 2.5. However, since the CYPRI Score is composed exclusively of whole-number criteria, the threshold was pragmatically set at ≥3. This cut-off yielded a sensitivity of 76% and a specificity of 57%. Consequently, the results of the Frankfurt cohort indicate patients with a CYPRI score of 3 or above are highly likely to benefit from clinically meaningful PGx test results. Additionally, ordinal logistic regression models were performed to assess the relationship between CYPRI scores and the degree of pharmacogenetic impact (IMPACT categories). In the Frankfurt cohort, each one-point increase in CYPRI_orig was associated with higher odds of a greater pharmacogenetic impact (OR = 1.27, 95% CI 1.08–1.48; *p* = 0.003), and a nearly identical result was observed for CYPRI_cor (OR = 1.27, 95% CI 1.08–1.48; *p* = 0.003). In the Prague cohort, the association was more pronounced (CYPRI_orig: OR = 1.81, 95% CI 1.26–2.75; *p* = 0.0027; CYPRI_cor: OR = 2.09, 95% CI 1.39–3.29; *p* = 0.0007). These results consistently demonstrate that patients with higher CYPRI scores have markedly increased odds of experiencing a clinically relevant pharmacogenetic impact.

### 3.3. Modification of the CYPRI Score: Results Using CYPRI_cor and IMPACT_orig Score in the Frankfurt Cohort

**Figure 3:**
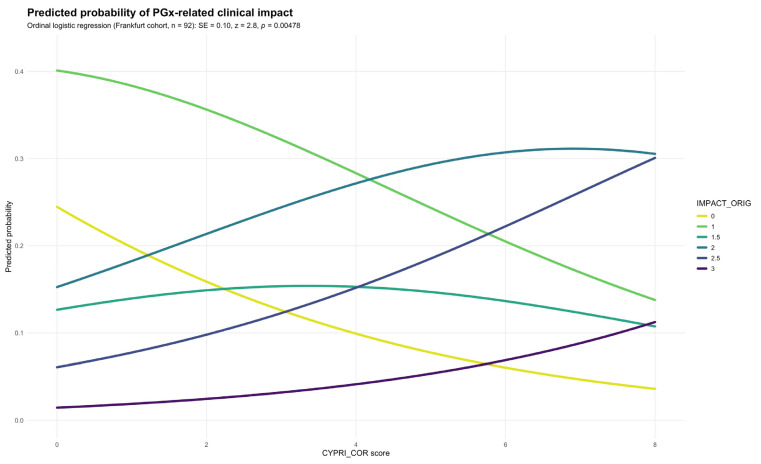
Predicted probability of IMPACT_orig by CYPRI_cor in the Frankfurt cohort (n = 92).

To evaluate the impact of the revised scoring criteria, the CYPRI_cor was applied to the Frankfurt cohort alongside the IMPACT_orig. Logistic regression showed a statistically significant positive association between CYPRI_cor and IMPACT_orig, indicating, as previously observed, that higher CYPRI_cor scores predicted more substantial PGx interventions. This association was supported by a standard error = 0.10 and a *p*-value of 0.00478 (Figure 3).

In contrast to the original model, the ROC analysis revealed a slightly lower discriminative performance, with an AUC of 0.68 (95% CI 0.56–0.79; Figure 2). While this still reflects a modest ability of the CYPRI_cor to distinguish patients most likely to benefit from PGx testing, it does not reach the same level of discriminative performance as observed with the CYPRI_orig score. Nonetheless, CYPRI_cor offers improved clinical specificity (69% vs. the previous 57%) by including a more selective interpretation of ADRs, focusing only on those plausibly linked to pharmacokinetic variability, as well as a refined categorization of TDM findings.

The optimal CYPRI cut-off was determined to be 2.5. Due to the CYPRI score resulting only in whole numbers, the Frankfurt cohort indicates a cut-off at 3, resulting in a sensitivity of 56% and a specificity of 69%.

### 3.4. Results Using the CYPRI_cor and IMPACT_orig Score Variants in the Prague Cohort

As a reference within the Prague cohort, we first applied the original scoring pair (CYPRI_orig with IMPACT_orig) to enable a direct, within-cohort comparison with the corrected score. This analysis showed a significant positive association (SE = 0.20; *p* = 0.0267), indicating that higher CYPRI_orig values were linked to greater medication impact.

**Figure 4:**
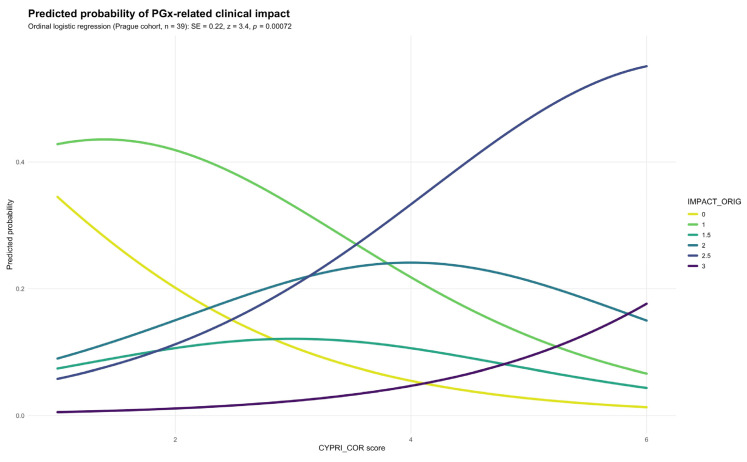
Predicted probability of IMPACT_orig by CYPRI_cor in the Prague cohort (n = 39).

Subsequently, CYPRI_cor was applied to the same patient cohort and yielded markedly stronger results, showing a highly significant association with the outcome measure IMPACT_orig (SE = 0.22; *p* = 0.00072), suggesting that the revised score maintains and potentially strengthens the relationship between CYPRI and clinical impact (Figure 4) compared to the original model (*p* = 0.0267). This reinforces the potential clinical value of the refined scoring approach.

**Figure 5:**
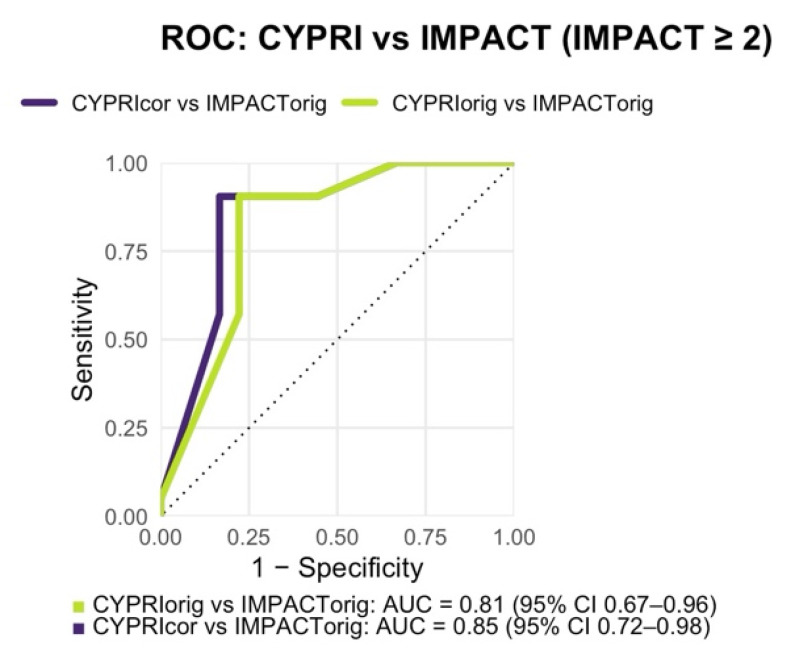
Receiver Operating Characteristic (ROC) analysis comparing CYPRI_orig vs. IMPACT_orig (AUC = 0.81, 95% CI 0.67–0.96) and CYPRI_cor vs. IMPACT_orig (AUC = 0.85, 95% CI 0.72–0.98) in the Prague cohort (n = 39).

With a resulting AUC of 0.85, the ROC analysis also indicated slightly better discriminative ability than the original model (AUC = 0.85 vs. 0.81; Figure 5).

The optimal cut-off in the Prague cohort, determined by Youden’s index, was 3.5, resulting in a sensitivity of 0.90 and specificity of 0.83. Because the CYPRI score uses whole numbers, this was rounded to a practical clinical threshold of 4 for subsequent analyses and recommendations.

## 4. Discussion

The study supports the CYPRI scoring system as a practical tool to identify mental health patients most likely to benefit from PGx testing, especially in relation to CYP2D6 and CYP2C19. In both cohorts, higher CYPRI Scores correlate significantly with greater clinical impact of PGx test results, as demonstrated in the ordinal regression. The ordinal regression indicated an association between the CYPRI score and the degree of pharmacogenetic impact across both cohorts. In the Frankfurt cohort, each one-point increase in CYPRI_orig was associated with higher odds of a greater PGx impact (OR = 1.27, 95% CI 1.08–1.48; *p* = 0.003), with a similar effect for CYPRI_cor (OR = 1.27, 95% CI 1.08–1.48; *p* = 0.003). The association was even stronger in the Prague cohort (CYPRI_orig: OR = 1.81, 95% CI 1.26–2.75; *p* = 0.0027; CYPRI_cor: OR = 2.09, 95% CI 1.39–3.29; *p* = 0.0007), confirming the consistency of the observed trend across datasets.

The IMPACT score reflects the clinical relevance of PGx testing by capturing *CYP2D6/CYP2C19* phenotype alterations and whether clinical management requires intensified monitoring (and related actions such as dose or medication changes). The ROC analysis further supported the association between CYPRI and IMPACT by quantifying its discriminative performance. In the Frankfurt cohort, the CYPRI_orig demonstrated acceptable discriminative ability (AUC = 0.71), whereas the CYPRI_cor version showed marginally lower AUC (0.68), indicating a minor reduction in discriminatory performance. Despite this, statistical significance was maintained because the regression coefficient for CYPRI_cor remained significant (ordinal logistic regression: SE = 0.10, *p* = 0.00478), indicating a monotonic association across the score range, even if threshold-based discrimination (AUC) decreased. Although the overall AUC changed minimally, the corrected CYPRI_cor achieved higher specificity (69% vs. the previous 57%), which represents an essential feature in clinical application, allowing for more accurate identification of patients who are truly likely to benefit from PGx testing. This specificity-first trade-off is clinically deliberate in resource-limited settings: prioritizing fewer false positives reduces unnecessary PGx tests and concentrates testing on patients with a higher pre-test probability of actionable findings. Thereby, we avoid costly over-testing.

The application of the CYPRI_cor on the Prague cohort achieved a better discrimination (AUC = 0.85 vs. 0.81), showing that direct patient contact in the form of face-to-face interviews and exploring the medication history is important to identify patients who are most likely to benefit from testing rather than retrospective data analysis as performed in Frankfurt. Furthermore, the ordinal regression confirmed that the Prague cohort demonstrated the strongest and most statistically significant association between the CYPRI score and pharmacogenetic impact, particularly for the corrected version (CYPRI_cor: OR = 2.09, 95% CI 1.39–3.29; *p* = 0.0007). This finding indicates that the corrected CYPRI model offers a more consistent performance across cohorts. In the Prague cohort, the Youden optimum was 3.5. Because CYPRI uses whole numbers, we round to a practical threshold of 4. Although the Frankfurt cohort indicated an optimum near 3 (Youden = 2.5), we adopt ≥4 as the unified clinical cut-off to prioritize specificity, avoid unnecessary testing, and target PGx more selectively. This should be confirmed by studies in other health care settings and in other health care systems. This choice is supported by the Prague cohort, in which prospective, in-hospital assessments with full access to medical records yielded more reliable data. Accordingly, ROC analysis showed a higher AUC and stronger statistical significance than in the retrospective Frankfurt cohort [18]. Although Youden’s index varied slightly across samples, a threshold of 4 provides a reasonably consistent cut point criterion for future application.

Although pharmacological treatment strategies guided by pre-emptive PGx testing have been shown to improve patient outcomes, the routine implementation of such testing in German hospitals remains limited due to the cost [7]. Until now, no systematic approach existed to reliably identify patients who would benefit most from genetic testing. The CYPRI score provides a tool that enables the selection of patients who benefit the most from PGx testing. Such genotype-informed treatment has the potential to enhance clinical outcomes, reflected not only in reduced length of hospital stay, but also in fewer adverse drug reactions, reduced treatment costs, and the prevention of treatment resistance [13].

The CYPRI_cor score was designed to optimize and rule out potential limitations of the original score, particularly regarding the attribution of ADRs and the weighting of TDM results. Although the modifications did not improve discriminative performance in the Frankfurt dataset, the proposed changes remain meaningful. Scoring of the CYPRI should only consider ADRs clearly related to drug concentrations, excluding those without such a link (e.g., rash) [26]. Moreover, the adjustments to the TDM scoring allow for a stronger weighting of plasma concentrations outside the recommended range, thereby placing greater emphasis on the therapeutic efficacy of the medications.

To demonstrate the rationale behind the proposed modifications, the CYPRI_cor was applied to the extended original Prague dataset from the first publication of the CYPRI score, after consultation with the original developer of the score [18]. In this analysis, the corrected score achieved stronger and statistically more robust results (*p* = 0.00072 vs. 0.267), thereby supporting the suggested refinements. The discrepancy observed in the Frankfurt cohort may be attributed to the retrospective nature of the data collection in Frankfurt. Specifically, the CYPRI scores were derived from discharge summaries and physicians’ documentation retrospectively, versus active scoring during hospitalization in the Prague cohort, where information could have been retrieved from ongoing records or supplemented interviews. That represents a limitation of the study. Another limitation lies in the subjective assessment of certain CYPRI criteria. ADRs and treatment resistance rely heavily on the clinical judgment of physicians, which complicates standardization. The proposed refinements to the ADR criteria represent an initial step toward minimizing this source of bias, though complete elimination remains impossible. Furthermore, the collaboration with the original developer of the CYPRI Score could lead to investigator bias. While ensuring methodological accuracy, bias could be introduced by the originator’s perspective, unconsciously influencing interpretations and refinements. Due to the limited sample size, we did not perform full cross-validation across folds. Instead, internal validation was conducted by non-parametric bootstrapping (1000 resamples) for the main model (Frankfurt cohort, CYPRI_cor IMPACT_orig), which demonstrated comparable performance and acceptable internal stability.

This study and the CYPRI framework are metabolism-focused. Genotyping was limited to *CYP2D6* and *CYP2C19*, i.e., CYP genes with established, guideline-based dosing recommendations for antidepressants and antipsychotics.

Future research should focus on validating the modified CYPRI score (the original score will not be used anymore; CYPRI is there for the term for the modified version in future publications) in diverse mental health populations, refining the ADR component to reduce inter-rater variability, and exploring integration into clinical workflows. These findings should be replicated in prospective, multicenter studies with larger samples drawn from different regions and healthcare systems worldwide. In addition, economic analyses could quantify the potential cost savings from targeted PGx testing guided by the CYPRI score. For clarity and consistency, the term “CYPRI” will henceforth refer to the corrected scale (formerly CYPRI_cor). The original designation will no longer be used in future publications and applications.

CYPRI remains in an early stage of development. Accordingly, integrating generative or explainable AI for patient selection or automated interpretation is not yet applicable to this rule-based score and lies beyond the scope of this study. Such approaches may be explored in future studies.

## 5. Conclusions

This study suggests the CYPRI Score as a potentially useful tool for identifying mental health patients who are most likely to benefit from PGx-testing. Higher CYPRI scores were associated with more frequent and substantial medication adjustments, indicating its utility as a guide for targeted testing strategies and optimizing antidepressant therapy. By integrating genetic information into clinical decision-making, the CYPRI Score has the potential to improve treatment outcomes, reduce adverse drug reactions, and support a more personalized and cost-effective approach to psychiatric pharmacotherapy. Given the retrospective design and limited sample size, further research in larger populations and a prospective study design is necessary to confirm these findings and explore their applicability in clinical practice and validate CYPRI across diverse patient populations and care settings.

## Figures and Tables

**Table 1:** CYPRI_orig Scoring Criteria and Point Allocation.

POINTS	CRITERIA	DEFINITION
1	Use of psychiatric medication with a high risk of CYP2C19/CYP2D6 metabolism variability	Psychiatric drugs classified as CYP2D6/CYP2C19 substrates based on: Flockhart Table [22], Hiemke 2018 Consensus Guidelines for Therapeutic Drug Monitoring (TDM) in Psychiatry [23], CPIC [16], and DPWG guidelines for PGx-based drug metabolism impact [17]. Examples: atomoxetine, modafinil, amitriptyline, nortriptyline, paroxetine, es-/citalopram, fluoxetine, aripiprazole, risperidone, diazepam, venlafaxine.
1	Use of somatic medication with a high risk of CYP2C19/CYP2D6 metabolism variability	Non-psychiatric drugs that are also CYP2D6/CYP2C19 substrates based on Flockhart Table, CPIC, and DPWG. Examples: clopidogrel, codeine, tramadol, omeprazole, pantoprazole, ondansetron, metoclopramide, voriconazole, metoprolol, propafenone.
1	Treatment-resistant condition despite confirmed adherence and adequate dosing.	Defined as failure of two or more adequate medication trials [24].
3	Drug plasma levels do not correspond to the administered dose	Based on TDM results, defined as follows: ▪ High dose with subtherapeutic drug levels (suggesting ultra-/rapid metabolism). ▪ Low dose with supratherapeutic/toxic drug levels (suggesting intermediate/poor metabolism). ▪ Plasma drug concentration falls outside the established minimum-maximum range (C/Dl_ow_ and C/D_high_) for the specific drug according to Hiemke 2018 Consensus Guidelines for Therapeutic Drug Monitoring in Psychiatry. ▪ Altered parent drug/metabolite ratio, indicating non-normal CYP2D6/CYP2C19 metabolism (e.g., venlafaxine, risperidone, aripiprazole). Note: Clinically relevant interaction/phenoconversion must be ruled out.
3	Clinically relevant adverse drug reactions (ADRs) from medications listed above	Based only on clinical observation and pharmacological anamnesis, defined as ADR: ▪ Requiring dose adjustment. ▪ Increased monitoring. ▪ Drug discontinuation due to intolerance or toxicity. ▪ Hospitalization due to ADR. ▪ Information that patient is unable to tolerate the drug despite standard dosing (reliable information in pharmacologic anamnesis, e.g., medical opinion).
9	Total Score	The sum of all criteria determines patient eligibility for PGx testing.

**Table 2:** CYPRI_cor Scoring Criteria and Point Allocation.

POINTS	CRITERIA	DEFINITION
1	Use of psychiatric medication with a high risk of CYP2C19/CYP2D6 metabolism variability	Psychiatric drugs classified as CYP2D6/CYP2C19 substrates based on: Flockhart Table [22], Hiemke 2018 Consensus Guidelines for Therapeutic Drug Monitoring (TDM) in Psychiatry [23], CPIC [16], and DPWG guidelines for PGx-based drug metabolism impact [17]. Examples: atomoxetine, modafinil, amitriptyline, nortriptyline, paroxetine, es-/citalopram, fluoxetine, aripiprazole, risperidone, diazepam, venlafaxine.
1	Use of somatic medication with a high risk of CYP2C19/CYP2D6 metabolism variability	Non-psychiatric drugs that are also CYP2D6/CYP2C19 substrates based on Flockhart Table, CPIC, and DPWG. Examples: clopidogrel, codeine, tramadol, omeprazole, pantoprazole, ondansetron, metoclopramide, voriconazole, metoprolol, propafenone.
1	Treatment-resistant condition despite confirmed adherence and adequate dosing.	Defined as failure of two or more adequate medication trials [24].
	Drug plasma levels do not correspond to the administered dose	Based on TDM results, defined as follows: (Clinically relevant interaction/phenoconversion must be ruled out using the Flockhart interaction tables as reference [22]; score according to the highest applicable category, only the highest sub score is awarded, no cumulative points)
3		▪ High dose with subtherapeutic drug levels (suggesting ultra-/rapid metabolism. ▪ Low dose with supratherapeutic/toxic drug levels (suggesting intermediate/poor metabolism).
1		▪ Altered parent drug/metabolite ratio, indicating non-normal CYP2D6/CYP2C19 metabolism (e.g., venlafaxine, risperidone, aripiprazole).
1		▪ Plasma level is not available, but calculated dose-corrected ratio (DCR) is out of recommended range [23].
3	Clinically relevant, drug-related adverse drug reactions (ADRs) from medications listed above	Based only on clinical observation and pharmacological anamnesis. Score 3 pts only for ADRs plausibly related to drug concentration (see [25,26]) and meeting ≥1 of the following: ▪ Requiring dose adjustment. ▪ Increased monitoring. ▪ Drug discontinuation due to intolerance or toxicity. ▪ Hospitalization due to adverse drug reaction. ▪ Information that patient is unable to tolerate the drug despite standard dosing (reliable information in pharmacologic anamnesis, e.g., medical opinion).
9	Total Score	The sum of all criteria determines patient eligibility for PGx testing.

**Table 3:** IMPACT_orig Scoring Criteria and Point Allocation.

POINTS	CRITERIA	DEFINITION
0	No impact	Patient is a normal metabolizer for CYP2D6 and CYP2C19; no immediate intervention required.
1	Minor impact	Patient is a non-normal metabolizer in CYP2D6 and/or CYP2C19, but no intervention is necessary based on the current medication regimen.
2	Major impact	Patient is a non-normal metabolizer in CYP2D6 and/or CYP2C19, requiring clinical action such as medication change, dose adjustment, or enhanced monitoring to prevent treatment failure or adverse effects.
+0.5	Dual enzyme alteration	The patient is a non-normal metabolizer in both CYP2D6 and CYP2C19, which may involve either decreased or increased enzyme function, leading to a more complex pharmacogenetic impact.
+0.5	Multiple medication adjustment	An action was required in more than two medications.

## Data Availability

The data presented in this study are available on request from the corresponding author. The data are anonymized patient datasets from the University Hospital Frankfurt and cannot be made publicly available due to institutional and ethical restrictions.

## Abbreviations

The following abbreviations are used in this manuscript:

PGx	Pharmacogenetic testing
CYPRI	CYP Pharmacogenetic Risk Score
ADRs	Adverse drug reactions
TDM	Therapeutic drug monitoring
CYPRI_cor	Corrected version of the CYPRI Score
CNV	Copy Number Variants
SNP	Single Nucleotide Polymorphisms
MDD	Major Depressive Disorder
CPIC	Clinical Pharmacogenetics Implementation Consortium
PharmGKB	Pharmacogenomics Knowledge Base
IM	Intermediate metabolizer
PM	Poor metabolizer
UM	Ultra-rapid metabolizer
RM	Rapid metabolizer
ROC	Receiver Operating Characteristic
AUC	Area under the curve
DPWG	Dutch pharmacogenetic working group
